# Supplementary material for: Small mammalian herbivores at moderate densities facilitate livestock growth by improving vegetation composition in grasslands
Source: eLife. 2026 Sep 8;15:RP111340. doi: 10.7554/eLife.111340 (PMC13553068; doi:10.7554/eLife.111340)
Supplement: Supplementary file 1 [file elife-111340-mdarchecklist1.docx]

**Materials Design Analysis Reporting (MDAR)**

**Checklist for Authors**

The [MDAR framework](https://osf.io/xfpn4/) establishes a minimum set of requirements in transparent reporting mainly applicable to studies in the life sciences.

*eLife* asks authors to **provide detailed information within their article** to facilitate the interpretation and replication of their work. Authors can also upload supporting materials to comply with relevant reporting guidelines for health-related research (see [EQUATOR Network](http://www.equator-network.org/%20)), life science research (see the [BioSharing Information Resource](http://biosharing.org/)), or animal research (see the [ARRIVE Guidelines](http://www.plosbiology.org/article/info:doi/10.1371/journal.pbio.1000412) and the [STRANGE Framework](https://doi.org/10.1038/d41586-020-01751-5); for details, see *eLife*’s [Journal Policies](https://reviewer.elifesciences.org/author-guide/journal-policies)). Where applicable, authors should refer to any relevant reporting standards materials in this form.

For all that apply, please note **where in the article** the information is provided. Please note that we also collect information about data availability and ethics in the submission form.

**Materials:**

| **Newly created materials** | **Indicate where provided: section/figure legend** | **N/A** |
| --- | --- | --- |
| The manuscript includes a dedicated "materials availability statement" providing transparent disclosure about availability of newly created materials including details on how materials can be accessed and describing any restrictions on access. |  | N/A |
|  |  |  |
| **Antibodies** | **Indicate where provided: section/figure legend** | **N/A** |
| For commercial reagents, provide supplier name, catalogue number and [RRID](https://scicrunch.org/resources), if available. |  | N/A |
|  |  |  |
| **DNA and RNA sequences** | **Indicate where provided: section/figure legend** | **N/A** |
| Short novel DNA or RNA including primers, probes: Sequences should be included or deposited in a public repository. |  | N/A |
|  |  |  |
| **Cell materials** | **Indicate where provided: section/figure legend** | **N/A** |
| Cell lines: Provide species information, strain. Provide accession number in repository OR supplier name, catalog number, clone number, OR RRID. |  | N/A |
| Primary cultures: Provide species, strain, sex of origin, genetic modification status. |  | N/A |
|  |  |  |
| **Experimental animals** | **Indicate where provided: section/figure legend** | **N/A** |
| Laboratory animals or Model organisms: Provide species, strain, sex, age, genetic modification status. Provide accession number in repository OR supplier name, catalog number, clone number, OR RRID. |  | N/A |
| Animal observed in or captured from the field: Provide species, sex, and age where possible. | Species, sex, age, and source (for wild-caught or observed animals):  Yaks (Bos grunniens) – sex: steers (castrated males); age: 2 years old; source: purchased from adjacent pastures in the study region.  Plateau pikas (Ochotona curzoniae) – observed and temporarily captured from the wild; sex and age were not recorded (captured individuals were used for short-term feeding observations and then released; in the manipulative experiment, pikas were removed from plots without individual identification).  Indicate where provided:  Yak details: Methods section, subsection “Field manipulative experiment” → “Experimental design” (e.g., “32 yak steers aged 2 years and weighing 115 ± 7.8 kg”).  Pika details: Methods section, subsections “Field survey #1: Diet preferences of pikas and yaks” (capture and release) and “Pika treatments” (removal procedures).  Study site and species background: Methods section, subsection “Study system and background”. |  |
|  |  |  |
| **Plants and microbes** | **Indicate where provided: section/figure legend** | **N/A** |
| Plants: provide species and strain, ecotype and cultivar where relevant, unique accession number if available, and source (including location for collected wild specimens). | Wild grassland plants (e.g., Kobresia humilis, Elymus nutans, and Stellera chamaejasme). No cultivars or accessions. All collected from the study site in Menyuan County, Qinghai, China (see Methods: Study system and background). |  |
| Microbes: provide species and strain, unique accession number if available, and source. |  | N/A |
|  |  |  |
| **Human research participants** | **Indicate where provided: section/figure legend) or state if these demographics were not collected** | **N/A** |
| If collected and within the bounds of privacy constraints report on age, sex, gender and ethnicity for all study participants. |  | N/A |

**Design:**

| **Study protocol** | **Indicate where provided: section/figure legend** | **N/A** |
| --- | --- | --- |
| If the study protocol has been pre-registered, provide DOI. For clinical trials, provide the trial registration number OR cite DOI. |  | N/A |
|  |  |  |
| **Laboratory protocol** | **Indicate where provided: section/figure legend** | **N/A** |
| Provide DOI OR other citation details if detailed step-by-step protocols are available. |  | N/A |
|  |  |  |
| **Experimental study design (statistics details) *** | | |
| **For in vivo studies: State whether and how the following have been done** | **Indicate where provided: section/figure legend. If it could have been done, but was not, write “not done”** | **N/A** |
| Sample size determination | Not done, Methods: "Field manipulative experiment" → "Experimental design" |  |
| Randomisation | Done, Methods: "Field manipulative experiment" → "Experimental design" → "Plot treatments were randomly assigned within each block" and "randomly grouped them... randomly allocated these yak herds to the 16 experimental plots". |  |
| Blinding | Not done | N/A |
| Inclusion/exclusion criteria | Done (implicity), Methods: "Field manipulative experiment" → "Experimental design" → "To avoid edge effects, we sampled... within the 100 × 100 m area at the center of each plot". |  |
|  |  |  |
| **Sample definition and in-laboratory replication** | **Indicate where provided: section/figure legend** | **N/A** |
| State number of times the experiment was replicated in the laboratory. |  | N/A |
| Define whether data describe technical or biological replicates. | Biological replicates.  For the field experiment (yak weight gain, plant cover, foraging efficiency), the replicates are biological: 4 spatial blocks (each containing 4 treatments) × 2 growing seasons (2022–2023) = 8 independent biological replicates per treatment.  For forage quality (nutritional values), the 5 individual forage samples collected per plot represent biological replicates, which were pooled (1:1 dry mass ratio) before laboratory analysis. The lab measurements themselves reflect biological variation across plots and years, not technical replication from repeated instrument runs.  Indicate where provided:  Methods: "Field manipulative experiment" → "Experimental design" (describes the 4 replicate blocks and 16 plots); Methods: "Forage quantity and quality" (describes collection of 5 samples per plot and pooling for lab analysis); Appendix 1—table 13 (summarises the statistical model structure with block, year, and month as random effects, confirming biological replication structure). Figure legends also refer to "combined effects of two-year (2022–2023)" experiments, indicating temporal replication. |  |
|  |  |  |
| **Ethics** | **Indicate where provided: section/submission form** | **N/A** |
| Studies involving human participants: State details of authority granting ethics approval (IRB or equivalent committee(s), provide reference number for approval. |  | N/A |
| Studies involving experimental animals: State details of authority granting ethics approval (IRB or equivalent committee(s), provide reference number for approval. | Methods section: "Animal welfare and ethics" , "All procedures involving live animals were approved by the Science and Technology Ethics Committee of Northeast Normal University, Changchun, China." |  |
| Studies involving specimen and field samples: State if relevant permits obtained, provide details of authority approving study; if none were required, explain why. | Methods section, first paragraph under "Methods" , "All pika and yak manipulations were carried out in accordance with the Law of the People's Republic of China on the Protection of Wildlife (1988)." |  |
|  |  |  |
| **Dual Use Research of Concern (DURC)** | **Indicate where provided: section/submission form** | **N/A** |
| If study is subject to dual use research of concern regulations, state the authority granting approval and reference number for the regulatory approval. |  | N/A |

**Analysis:**

| **Attrition** | **Indicate where provided: section/figure legend** | **N/A** |
| --- | --- | --- |
| Describe whether exclusion criteria were pre-established. Report if sample or data points were omitted from analysis. If yes, report if this was due to attrition or intentional exclusion and provide justification. | To avoid edge effects, sampling of all plant, pika, and yak variables was restricted to the central 100 × 100 m core area of each plot; the peripheral 50‑m buffer zone was pre‑excluded. In addition, only yaks of uniform age (2 years) and similar body weight (115 ± 7.8 kg) were selected as inclusion criteria at the start of the experiment.  See these information in Methods: "Field manipulative experiment" → "Experimental design" – "To avoid edge effects, we sampled plant, pika, and yak variables within the 100 × 100 m area at the center of each plot"; and "32 yak steers aged 2 years and weighing 115 ± 7.8 kg". |  |
|  |  |  |
| **Statistics** | **Indicate where provided: section/figure legend** | **N/A** |
| Describe statistical tests used and justify choice of tests. | We used linear models, GLMMs, and GAMs with random effects (block, year, month) to handle hierarchical data. Distributions were chosen based on response variables: Gaussian for continuous data, Beta for proportions, and Tweedie for zero‑inflated/non‑normal data (e.g., bite rates), as a Gaussian fit was poor. GAMs were used for non‑linear relationships. Post‑hoc Tukey‑HSD or Sidak tests were applied only when the pika × Stellera interaction was significant. All analyses used R packages glmmTMB, mgcv, and DHARMa (for diagnostics).  See these information in Methods: "Statistical analyses"; full model summary in Appendix 1—table 13. |  |
|  |  |  |
| **Data availability** | **Indicate where provided: section/submission form** | **N/A** |
| For newly created and reused datasets, the manuscript includes a data availability statement that provides details for access (or notes restrictions on access). | The manuscript contains a dedicated "Data availability" section that states all data are publicly accessible and provides both a Zenodo DOI and a GitHub link. No restrictions on access are mentioned.  See these information in Data availability section (immediately before References). |  |
| When newly created datasets are publicly available, provide accession number in repository OR DOI and licensing details where available. | DOI: 10.5281/zenodo.18290921  Repository: Zenodo (also mirrored on GitHub: https://github.com/ddlawton/pika_yak_interactions)  Licensing details: Not specified in the manuscript (the repository may contain a license; if required, we can state "not provided in the manuscript" or "no specific license stated").  See these information in Data availability section – the DOI and GitHub link are explicitly given. |  |
| If reused data is publicly available provide accession number in repository OR DOI, OR URL, OR citation. |  | N/A |
|  |  |  |
| **Code availability** | **Indicate where provided: section/figure legend** | **N/A** |
| For any computer code/software/mathematical algorithms essential for replicating the main findings of the study, whether newly generated or re-used, the manuscript includes a data availability statement that provides details for access or notes restrictions. | The manuscript includes a clear statement that all R code used for data management, statistical modeling, and visualisation is publicly accessible. No restrictions on access are mentioned.  See these information in Data availability section (before References). Also mentioned in the Methods: "Statistical analyses" subsection: "All data management, modeling, and visualization approaches were carried out in R, with dependencies managed using renv. ... A complete record of package versions is available in the renv.lock file in the repository Zenodo: https://doi.org/10.5281/zenodo.18290921." |  |
| Where newly generated code is publicly available, provide accession number in repository, OR DOI OR URL and licensing details where available. State any restrictions on code availability or accessibility. | DOI: 10.5281/zenodo.18290921  URL (GitHub): https://github.com/ddlawton/pika_yak_interactions  Licensing details: Not specified in the manuscript. The repository may include a license; if not stated, we can write "Licensing details not provided in the manuscript".  Restrictions: None – the code is freely accessible without any restrictions.  See these information in Data availability section provides the DOI and GitHub link. The manuscript does not mention licensing or restrictions. |  |
| If reused code is publicly available provide accession number in repository OR DOI OR URL, OR citation. |  | N/A |

**Reporting:**

The MDAR framework recommends adoption of discipline-specific guidelines, established and endorsed through community initiatives.

| **Adherence to community standards** | **Indicate where provided: section/figure legend** | **N/A** |
| --- | --- | --- |
| State if relevant guidelines (e.g., ICMJE, MIBBI, ARRIVE, STRANGE) have been followed, and whether a checklist (e.g., CONSORT, PRISMA, ARRIVE) is provided with the manuscript. | ARRIVE guidelines followed (implicitly). This is an animal study involving live yaks and plateau pikas. The manuscript reports essential ARRIVE items including: ethical approval (Science and Technology Ethics Committee of Northeast Normal University), study design (2×2 factorial field experiment with replication), sample characteristics (species, sex, age), randomisation (treatments randomly assigned within blocks), blinding (not done – explained as not feasible in field conditions), sample size (logistically determined, no power analysis), inclusion/exclusion criteria (age/weight selection; central core sampling), statistical methods (detailed with justification), and housing/husbandry (yaks grazed daily and housed overnight; pikas trapped and relocated). No ARRIVE checklist is provided as a separate supplementary file.  See these information in Ethics approval: Methods: "Animal welfare and ethics". Experimental design: Methods: "Field manipulative experiment" → "Experimental design"; Appendix 1—table 13. Animal details: Methods: "Study system and background" and "Experimental design". Randomisation: Methods: "Experimental design". Blinding: Not stated in manuscript (explicitly "not done" in MDAR). Sample size: Not formally determined (stated as "not done" in MDAR). Inclusion/exclusion: Methods: "Experimental design". Statistics: Methods: "Statistical analyses"; Appendix 1—table 13. |  |

* We provide the following guidance regarding transparent reporting and statistics; we also refer authors to [Ten common statistical mistakes to watch out for when writing or reviewing a manuscript](https://doi.org/10.7554/eLife.48175).

**Sample-size estimation**

- You should state whether an appropriate sample size was computed when the study was being designed
- You should state the statistical method of sample size computation and any required assumptions
- If no explicit power analysis was used, you should describe how you decided what sample (replicate) size (number) to use

**Replicates**

- You should report how often each experiment was performed
- You should include a definition of biological versus technical replication
- The data obtained should be provided and sufficient information should be provided to indicate the number of independent biological and/or technical replicates
- If you encountered any outliers, you should describe how these were handled
- Criteria for exclusion/inclusion of data should be clearly stated
- High-throughput sequence data should be uploaded before submission, with a private link for reviewers provided (these are available from both GEO and ArrayExpress)

**Statistical reporting**

- Statistical analysis methods should be described and justified
- Raw data should be presented in figures whenever informative to do so (typically when N per group is less than 10)
- For each experiment, you should identify the statistical tests used, exact values of N, definitions of center, methods of multiple test correction, and dispersion and precision measures (e.g., mean, median, SD, SEM, confidence intervals; and, for the major substantive results, a measure of effect size (e.g., Pearson's r, Cohen's d)
- Report exact p-values wherever possible alongside the summary statistics and 95% confidence intervals. These should be reported for all key questions and not only when the p-value is less than 0.05.

**Group allocation**

- Indicate how samples were allocated into experimental groups (in the case of clinical studies, please specify allocation to treatment method); if randomization was used, please also state if restricted randomization was applied
- Indicate if masking was used during group allocation, data collection and/or data analysis
