# Supplementary material for: Small mammalian herbivores at moderate densities facilitate livestock growth by improving vegetation composition in grasslands
Source: eLife. 2026 Sep 8;15:RP111340. doi: 10.7554/eLife.111340 (PMC13553068; doi:10.7554/eLife.111340)
Supplement: Peer review history

# Editor report: eLife Assessment

**DOI:** 10.7554/eLife.111340.3.sa0

**Authors:** Sergio Rasmann

**Affiliations:** University of Neuchâtel Switzerland

**Keywords:** Convincing, Important

This **important** study provides evidence that plateau pikas, at moderate densities, can facilitate yak nutrition by suppressing a poisonous plant, offering a helpful perspective on reciprocal interactions between small mammal ecosystem engineers and large herbivores. The evidence is **convincing**, supported by a manipulative field experiment and appropriate measurements of intermediary ecological processes. The work will be of interest to ecologists, conservation biologists, and rangeland managers, particularly those studying grassland herbivore interactions and livestock management.

# Referee report: Reviewer #1 (Public review)

**DOI:** 10.7554/eLife.111340.3.sa1

Summary:

This is important and significant work because it helps describe the complexity of interactions between system components where 2 herbivores interact with vegetation. Whereas other studies have shown that the larger ungulate (yaks, Bos grunniens, in this case) can facilitate the abundance and population growth of the smaller (the semi-fossorial lagomorph, Ochotona curzoniae, plateau pika hereafter), this study flips the tables, and shows that, at least under some conditions, moderate densities of the plateau facilitate the nutritional condition of yaks.

Strengths:

Notably, the strong inference the authors can claim for their results is supported by the careful experimental design. A weaker paper would have simply noted correlations between pika burrow density and yak feeding efficiency without experimental removal. This paper, to its credit, not only used experimental removals but also documented the various intermediary results that support the ultimate conclusions. The statistical approaches used appear to be appropriate. (Readers are encouraged to read the full Materials and Methods, which are available in the Supplementary Materials section).

Weaknesses:

Although the study was well designed and executed, and its conclusions appear strongly supported, readers interested in the management implications on the Qinghai-Tibetan Plateau should be mindful of its limitations. First, the study site, at approximately 3,200 m elevation, was relatively low by Qinghai-Tibetan Plateau standards. Stellera chamaejasme becomes less common at elevations > 4,000 m, where a majority of livestock grazing occurs. Thus, it would be instructive to learn, through follow-up studies, whether similar facilitation occurs where unpalatable (and mildly poisonous) species in such genera as Astragalus, Oxytropis, and Thermopsis replace S. chamaejasme as the problematic plant for pastoralists. Second, the authors make no mention of wild ungulates, so it is unclear what, if any, role they may have played in this system. At least one study in Qinghai Province, albeit at a slightly higher elevation, showed that not only pikas, but also Tibetan gazelles (Procapra picticaudata), which were commonly observed on grazed pastures, grazed more frequently on some dicots avoided by domestic sheep than did the livestock themselves (Harris et al. 2015). It would also be instructive to learn if similar facilitation as observed here applied to the other principal livestock species in the area, domestic sheep (which are often herded together with smaller numbers of domestic goats). Finally, as suggested by this study, the interactions between all components of the system are complex and interactive. If pika facilitation of yak nutrition at the densities documented results in herders increasing yak density, might the increased herbivory from the domestic animals provide the conditions for the pika population to increase beyond the densities observed here, and thus toward the levels where facilitation yields to competition?

# Referee report: Reviewer #2 (Public review)

**DOI:** 10.7554/eLife.111340.3.sa2

This study uses a combination of field sampling and manipulative experiments to test for facilitative impacts of pikas on yaks via suppression of a poisonous forb. The authors found that, when Stellera forbs were present, yak weight increases over the growing season were greater in the presence of pikas compared to in their absence. This occurred because, although pikas do not consume Stellera, they clip it and use it in nest/burrow construction, thereby decreasing its relative abundance in the plant community. Thus, overall, the study contributes to our understanding of how herbivores of different size classes indirectly affect each other via use of shared resources.

It is well known that large herbivores on grasslands impact smaller animals, but the reciprocal interaction is rarely tested. Thus, this study asks a valuable question, and the experiment is well-designed to test it. The authors also do a good of demonstrating the potential conservation impacts of their research.

# Author comment: Author response

**DOI:** 10.7554/eLife.111340.3.sa3

**Authors:** Zhiwei Zhong, Bingbo Ni, Douglas Lawton, Xiaofei Li, Xiaona Zheng, Huakun Zhou, Junhu Su, Wenjin Li, Fujiang Hou, Zhenggang Guo, Quanmin Dong, Shikui Dong, Christopher R Dickman, Jens-Christian Svenning, Ying Gao, Zhibin Zhang

**Affiliations:** Northeast Institute of Geography and Agroecology, Chinese Academy of Sciences Changchun China; Northeast Institute of Geography and Agroecology, Chinese Academy of Science Changchun China; Arizona State University Tempe United States; Jilin Agricultural University Changchun China; Northeast Normal University Changchun China; Northwest Institute of Plateau Biology Xining China; Gansu Agricultural University Lanzhou China; Lanzhou University Lanzhou China; Lanzhou University Lanzhou China; Lanzhou University Lanzhou China; Qinghai University Xining China; Beijing Forestry University Beijing China; The University of Sydney Sydney Australia; Aarhus University Aarhus Denmark; Northeast Normal University Changchun China; Hainan University Haikou China

The following is the authors’ response to the original reviews.

**eLife Assessment**

This important study provides evidence that plateau pikas, at moderate densities, can facilitate yak nutrition by suppressing a poisonous plant, offering a helpful perspective on reciprocal interactions between small mammal ecosystem engineers and large herbivores. The evidence is solid, supported by a manipulative field experiment and appropriate measurements of intermediary ecological processes, although some claims about density dependence, competition, and stress-gradient mechanisms are not fully supported by the experimental design. The work will be of interest to ecologists, conservation biologists, and rangeland managers, particularly those studying grassland herbivore interactions and livestock management on the Qinghai-Tibetan Plateau.

Thank you very much for these positive assessments of our work. Below, we provide point-by-point responses to the comments from the two peer reviewers, and we hope these revisions are satisfied by you and the reviewers.

**Reviewer #1 (Public review):**

Summary:

This is important and significant work because it helps describe the complexity of interactions between system components where two herbivores interact with vegetation. Whereas other studies have shown that the larger ungulate (yaks, Bos grunniens, in this case) can facilitate the abundance and population growth of the smaller (the semi-fossorial lagomorph, Ochotona curzoniae, plateau pika hereafter), this study flips the tables and shows that, at least under some conditions, moderate densities of the plateau facilitate the nutritional condition of yaks.

The study was not designed to investigate the reasons that pikas clip Stellera chamaejasme. That said, based on other studies and general knowledge of the ecology of these pikas, it is likely that they clip (although do not eat) this plant because its relatively large size hinders predator detection. This species of pika does better where vegetation height is low than where it is higher.

Strengths:

Notably, the strong inference the authors can claim for their results is supported by the careful experimental design. A weaker paper would have simply noted correlations between pika burrow density and yak feeding efficiency without experimental removal. This paper, to its credit, not only used experimental removals but also documented the various intermediary results that support the ultimate conclusions. The statistical approaches used appear to be appropriate. (Readers are encouraged to read the full Materials and Methods, which are available in the Supplementary Materials section.)

We appreciate these positive comments on our work.

Weaknesses:

Although the study was well designed and executed, and its conclusions appear strongly supported, readers interested in the management implications of the Qinghai-Tibetan Plateau should be mindful of its limitations. First, the study site, at approximately 3,200 m elevation, was relatively low by Qinghai-Tibetan Plateau standards. Stellera chamaejasme becomes less common at elevations > 4,000 m, where a majority of livestock grazing occurs. Thus, it would be instructive to learn, through follow-up studies, whether similar facilitation occurs where unpalatable (and mildly poisonous) species in such genera as Astragalus, Oxytropis, and Thermopsis replace S. chamaejasme as the problematic plant for pastoralists.

Thank you for this suggestion. We have acknowledged this limitation in the Discussion by adding the paragraph below (see the Third point):

“Despite of these, several questions deserve further investigation. First, our study examined pika–yak interactions only during the summer period, when food resources are most abundant. Whether such facilitative effects weaken or even shift toward competition under more stressful conditions—for example, when forage becomes limited during autumn or winter—remains to be tested. Second, if the documented facilitation of yak nutrition by pikas prompts herders to increase yak densities, could the resulting rise in livestock herbivory push pika populations beyond the levels observed here, potentially toward the threshold where facilitation gives way to competition? Third, our study site is located at approximately 3,200 m elevation, relatively low by Qinghai-Tibetan Plateau standards. *Stellera* becomes less common at elevations > 4,000 m, where a majority of livestock grazing occurs. It would be instructive to learn, through follow-up studies, whether similar facilitation occurs where unpalatable (and mildly poisonous) species in such genera as *Astragalus, Oxytropis*, and *Thermopsis* replace *Stellera* as the problematic plants for pastoralists (Lu et al., 2012; Li and Zhao, 2025). Finally, it is unclear whether similar facilitation as observed here applied to the other principal livestock species in the area, such as domestic sheep and goats.”

See these revisions in Line 272-286 in the Discussion section.

Second, the authors make no mention of wild ungulates, so it is unclear what, if any, role they may have played in this system. At least one study in Qinghai Province, albeit at a slightly higher elevation, showed that not only pikas, but also Tibetan gazelles (Procapra picticaudata), which were commonly observed on grazed pastures, grazed more frequently on some dicots avoided by domestic sheep than did the livestock themselves (Harris et al. 2015).

Citation:

Harris RB, Wang, WY, Badinqiuying , Smith AT, Bedunah DJ (2015) Herbivory and Competition of Tibetan Steppe Vegetation in Winter Pasture: Effects of Livestock Exclosure and Plateau Pika Reduction. PLoS ONE 10(7): e0132897. doi:10.1371/journal.pone.0132897

Thank you for this suggestion. We have added more details about the study site, particularly regarding wild ungulates, in the Methods section. Specifically, we have included the sentence of “Wild ungulates, such as Tibetan gazelles (*Procapra picticaudata*) (Harris et al., 2015), and other small mammals such as rabbits and zokors, occur rarely in the area.”

See these revisions in Line 333-335 in the Methods section.

It would also be instructive to learn if similar facilitation as observed here applied to the other principal livestock species in the area, domestic sheep (which are often herded together with smaller numbers of domestic goats).

Thank you for the suggestion. We have acknowledged this limitation in the Discussion, by adding a paragraph as: “Finally, it is unclear whether similar facilitation as observed here applied to the other principal livestock species in the area, such as domestic sheep and goats.”

See these revisions in Line 284-286 in the Discussion section.

Finally, as suggested by this study, the interactions between all components of the system are complex and interactive. If pika facilitation of yak nutrition at the densities documented results in herders increasing yak density, might the increased herbivory from the domestic animals provide the conditions for the pika population to increase beyond the densities observed here, and thus toward the levels where facilitation yields to competition?

Thank you for your suggestion. We have acknowledged this limitation in the Discussion, by adding the paragraph as “Second, if the documented facilitation of yaks by pikas prompts herders to increase yak densities, could the resulting rise in livestock herbivory push pika populations beyond the levels observed here, potentially toward the threshold where facilitation gives way to competition (Yang et al., 2026)?”

See these revisions in Line 276-279 in the Discussion section.

**Reviewer #1 (Recommendations for the authors):**

Although no doubt a bit sensitive, it would have been better to reveal a bit more about how pikas were removed.

We have provided more details about how pikas were removed in the no-pika treatment, by adding “For the no-pika treatment, pikas were trapped once every two weeks using 30 live traps (25 cm high × 25 cm wide × 40 cm long) within each plot and relocated elsewhere in the study site.” in the Methods section. We didn’t recorded how many pikas were removed from the corresponding plots, so no data were available for this point.

See these revisions in Line 411-413 in the Methods section.

The authors also missed a few relevant papers worth citing, including Badingqiuying, R. B. Harris, and A. T. Smith. 2018. Summer habitat use of plateau pikas (Ochotona curzoniae) in response to winter livestock grazing in the alpine steppe Qinghai-Tibetan Plateau. Arctic, Antarctic, and Alpine Research 50 (1): e1447190

We have cited this key paper in Line 75 in the Introduction section.

**Reviewer #2 (Public review):**

Summary:

This study uses a combination of field sampling and manipulative experiments to test for facilitative impacts of pikas on yaks via suppression of a poisonous forb. The authors found that, when Stellera forbs were present, yak weight increases over the growing season were greater in the presence of pikas compared to in their absence. This occurred because, although pikas do not consume Stellera, they clip it and use it in nest/burrow construction, thereby decreasing its relative abundance in the plant community. Thus, overall, the study contributes to our understanding of how herbivores of different size classes indirectly affect each other via the use of shared resources.

Strengths:

It is well known that large herbivores on grasslands impact smaller animals, but the reciprocal interaction is rarely tested. Thus, this study asks a valuable question, and the experiment is well-designed to test it. The authors also do a good job of demonstrating the potential conservation impacts of their research.

We appreciate these positive comments on our work.

Weaknesses:

What the authors tested is really cool, but their claims go far beyond what they can say based on their experimental design. For example, the authors claim to show that pika impacts on yaks display density-dependent transitions from competition to facilitation. However, their experiment only looked at the presence (at moderate densities) and absence of pikas, and they only tested for facilitation, not competition.

The paper would also benefit from changes to the framing in the introduction and discussion. For example, the authors pitch the work as a test of the stress-gradient hypothesis. However, there is no abiotic stress gradient in the study, which is an essential component of the SGH. They also pitch the work in terms of density dependence, but there is no significant variation in population densities beyond the presence-absence binary. The paper would be stronger if they focused their framing around the literature on facilitative interactions across mammals of different size classes, especially indirect facilitation via use of shared resources, which is what this paper is really about.

We agree that our work had explored only the facilitative effects of pikas on yaks, rather than the Stress Gradient Hypothesis (SGH). Thus, we deleted the description on SGH. However, the finding of a humped relation between yak weight gains and pika burrow densities (Figure 3C) is very important which provides evidence that moderate densities of pikas has the best beneficial effects on yak growth. We added a separate paragraph in discussion to have a clear discussion.

We have made the major revisions below to address these concerns.

(1) We have revised the title into “Small mammalian herbivores at moderate densities facilitate livestock growth by improving vegetation composition in grasslands ”.

(2) We have deleted all the statements about facilitation and competition predicted by the SGH in the Abstract (Line 56-59), Introduction (Line 88-91), Discussion (Line 231-233, the whole paragraph about SGH was removed here), and the References sections.

(3) We added a paragraph in discussion (Line 248-259) to have a clear discussion on the humped relation between yak weight gains and pika burrow densities as “Because of the natural variations in pika density in the pika-present treatment, we were able to obtain a hump-shaped relationship between yak weight gains and pika burrow densities in these plots. Compared with the absence of pikas, the facilitative effect reached its maximum at approximately 200 burrows/ha but became competitive at densities exceeding 400 burrows/ha (Figure 3C). This result reveals that pika density modulates the net outcome for yak weight gain, with a facilitation peak at ~200 burrows/ha and a competition onset above 400 burrows/ha. Our findings offer empirical evidence for the non-monotonicity theory, under which the competition-facilitation balance varies with population density: facilitation dominates at low densities, competition at high densities, and these density-dependent shifts may underpin community stability and productivity (Zhang, 2003; Zhang et al., 2015). The theory further holds that the facilitation threshold, not the competition-facilitation transition, is the critical factor governing the stability of interacting species or communities (Zhang et al., 2015).”

Most importantly, there are inconsistencies in what is visualized in the figures compared to the model results. For example, the results section in several places notes a lack of significant interaction terms in the model but shows interactions in the p-values on the figures.

In the Results section, there are only two places where we discussed non-significant interactions: Line 175–177 “Pikas and *Stellera* had no interactive effects on abundance of sedges, forbs, and neutral detergent fiber (NDF) of total forage for yaks (Figure 3F, I and Appendix 1—figure 1, table 5,8).” and Line 190–192 “Pikas and *Stellera* had no interactive effects on yaks’ foraging efficiency on forbs (Appendix 1—figure 2, table 10).”.

We have cross-checked both the Results section and the Figures sections mentioned above, and confirmed that they are consistent now.

The authors also plot smoothed lines rather than their model results and then draw interpretations from those lines that cannot be tested in the models that they used.

Thank you for the suggestion. Now we have added the Appendix 1—table 3 and Appendix 1—table 7 for the model results of generalized additive models (GAMs) for Figure 2C and Figure 3C that plotted with smoothed lines in Appendix 1.

There are also missing details that are important for model interpretation, including the distributions used and the sample sizes.

We have provided the Appendix 1—table 13 to summarize all statistical models used in the study, including the distributions used and the sample sizes in the Appendix 1.

We have also added a sentence of “A summary of all statistical models used in the study is available in Appendix 1 table 13.” in Line 475-476 in the Statistical analyses section to indicate this information.

Another major concern with experimental design is in the forage nutrient analyses. The authors picked plants along a grazing trail, then measured nutrient content without standardizing based on plant species, so any differences across treatments could be because of what they happened to grab rather than overall forage quality.

We have revised this section to provide more details on how forage samples were collected and their quality were analyzed. Specifically, five forage samples were collected per grazing plot, focusing on the two dominant plant species—one sedge and one grass—that were most frequently grazed by yaks. To ensure comparability across plots and treatments, we mixed the two species at equal dry mass (5 g). We have revised this section as below.

“To assess forage quality, five forage samples were collected from each grazing plot to quantify their nutritive values. To obtain samples that reflect the forage actually consumed by yaks, we tracked the animals along their grazing paths and collected the plant tissues of the two most frequently consumed species: the dominant sedge *Kobresia humilis* and the dominant grass *Elymus nutans* (Figure 2B; Pan et al., 2019). The collected tissues of each species were dried in a forced-air oven at 60 °C for 48 h, then ground through a 1-mm mesh. Subsequently, 5 g of each dried and ground species were combined in a 1:1 dry mass ratio, and the resulting mixture was stored in plastic bags for subsequent analyses.”

See these revisions in Line 439-447 in the Methods section.

**Reviewer #2 (Recommendations for the authors):**

(1) Introduction

Line 53 - I wouldn't describe small mammals like rodents as keystone species. They can have strong impacts on ecosystems, but not disproportionate relative to population size, which is a key part of that definition. It may be true when you talk specifically about pikas later on, but not small mammals as a general category.

We have replaced this term with “consumers” here, see Line 63.

Lines 58-61 - Good hook

Thank you for this positive comment.

Lines 69-74 - I don't think the stress gradient hypothesis is the right pitch for this work. The SGH posits that facilitation increases with abiotic stress, but no abiotic stressors were measured in this study. Population density interacts with abiotic factors in the SGH, but population density in and of itself is not an abiotic stressor. The papers you cite here all look at the interaction between population density and abiotic stressors (e.g., water availability). So these lines set me up to expect a stress gradient in your experiment that didn't exist, then left me confused later on. It would be better to highlight the strength of your work (lines 70-76 pose interesting questions and predictions) rather than trying to make it fit within the SGH.

Thank you for pointing out this problem. We have deleted the statements about SGH here.

See these revisions in Line 88-91 in the Introduction section.

(2) Materials and Methods

Lines 498-509 - Did you verify beforehand that no plants within the enclosures had been grazed on? How did you know that the consumption or clipping was specifically from those pikas?

We have clarified here by adding “Before cage installation, we carefully checked the plants within each plot and removed those that had been previously grazed or damaged by herbivores.” in the Methods section. In this case, we made it sure that the consumption or clipping was specifically from those pikas with the cages.

See the revisions in Line 349-350 in the Methods section.

Lines 509-512 - I would be careful calling this preference. It's really just a record of what they consumed along paths they were walking, which could be about accessibility and convenience as much as preference.

We have replaced “diet preferences” with “diet composition” here, see Line 359 in the Methods section.

Line 514 - Clarify the specific question or hypothesis you're testing with this field survey, beyond just generally testing associations.

We have modified the sentences here as “In July 2021, we investigated the potential facilitation of pikas on yaks mediated by the poisonous *Stellera* forbs under unmanipulated field conditions in the study site.”.

See Line 365-366 in the Methods section.

Lines 532-534 - The intro for this paper sets it up to be about density-dependent movement from competition to facilitation, but the experiment here is set up to compare pika presence/absence. The framing of the paper needs to be adjusted to better align with this experimental design.

The same issue as mentioned above. We agree that our work had explored only the facilitative effects of pikas on yaks, rather than the balance between competition and facilitation as predicted by the Stress Gradient Hypothesis (SGH). However, the finding of a humped relation between yak weight gains and pika burrow densities (Figure 3C) is very important which provides evidence that moderate density of pika has the best benefical effect on yak. We added an separate paragraph in discussion to have a clear discussion about this point in the Discussion section.

We have made the major revisions below to address this concern.

(1) We have revised the title as “Small mammalian herbivores at moderate densities facilitate livestock growth by improving vegetation composition in grasslands”.

(2) We have deleted the statements about facilitation and competition and the SGH in the Abstract (Line 56-59), Introduction (Line 88-91), Discussion (Line 231-233, the whole paragraph about SGH was removed here), and the References sections.

(3) We kept the discussion on the humped relation between yak weight gains and pika burrow densities (Figure 3C). We added an separate paragraph in discussion to have a clear discussion about this point. For details, see Line 248-259 in the Discussion section.

Lines 566-569 - Why did you need to simulate pika clipping when you already had pika presence/absence treatments?

We conducted *Stellera* removal treatment by simulating poisonous plant clipping behaviors of pikas because we want to confirm that the shifts in abundance of this dominant poisonous plant species is the key mechanism in driving pika-yak facilitation in our system. If we simply looked the differences in yak weight gain in the pika presence/absence treatments, it should be difficult to secure the underlying mechanism. In addition to the reduction in abundance of the poisonous *Stellera*, pikas may cause a variety of shifts vegetation properties including plant productivity and diversity, and soil disturbances that can exert direct and indirect effects on yak foraging activities, and thus their weight gains.

Lines 566-569 - Is there another citation you can give showing that Stellera forbs taller than 20cm are both preferred by pikas and exert greater impacts on plant/animal communities? Those are big assumptions that need to be better supported or explained. If there was a logistical reason that you didn't remove all of the smaller forbs, that needs to be laid out as well.

We have added one new citation here to support this method here. We have revised this section as “To simulate the clipping behavior of pikas, we clipped only those *Stellera* forbs exceeding 20 cm in height. This threshold was chosen based on previous observations that pikas preferentially target large forbs of this size (Liu et al., 2009).”

Liu W, Zhang Y, Wang X, Zhao JZ, Xu QM, Zhou L. 2009. The relationship of the harvesting behavior of plateau pikas with the plant community (In Chinese). Acta Theriologica Sinica 29:40-49.

Also, we have deleted the sentence of “and can exert significant impacts on the plant community and on yak grazing behaviors (Z.Z., field observations)” mentioned above, because these patterns were observed only by the authors in the field and lack supporting data.

See these revisions in Line 419-422 in the Methods section.

Line 587 - sample size per treatment? Was it consistently one species that you measured, and if so, which one? If you collected different species or a mix of species across treatments, then you can't really compare the nutrient values because you haven't accounted for interspecific variation.

We have revised this section to provide more details on how forage samples were collected and their quality were analyzed. Specifically, five forage samples were collected per grazing plot, focusing on the two dominant plant species—one sedge and one grass—that were most frequently grazed by yaks. To ensure comparability across plots and treatments, we mixed the two species at equal dry mass (5 g).

We have revised this section as below:

“To assess forage quality, five forage samples were collected from each grazing plot to quantify their nutritive values. To obtain samples that reflect the forage actually consumed by yaks, we tracked the animals along their grazing paths and collected the plant tissues of the two most frequently consumed species: the dominant sedge *Kobresia humilis* and the dominant grass *Elymus nutans* (Fig. 2B; Pan et al., 2019). The collected tissues of each species were dried in a forced-air oven at 60 °C for 48 h, then ground through a 1-mm mesh. Subsequently, 5 g of each dried and ground species were combined in a 1:1 dry mass ratio, and the resulting mixture was stored in plastic bags for subsequent analyses.”

See these revisions in Line 439-447 in the Methods section.

Lines 603-613 - Please provide the dependent variables in each model, as well as any interaction terms, in addition to the random effects. Please also state explicitly what you were trying to test with each of these models.

There are two models that use the tweedie family (forb and sedge bite rate). Indeed, we need to include the tweedie power parameter to help understand the mixture of the three families. We have included the p-value (power) in the Appendix 1—table 13 in the Appendix 1. We chose to use tweedie because a normal gaussian family model fitted the results poorly.

We have added the Appendix 1—table 13 in the Appendix 1, which provides the summary of all statistical models used in the study, including response variables, model type, distribution family (with Tweedie power parameter where applicable), interaction terms, random effects structure, and sample sizes.

Also, we have added a sentence of “A summary of all statistical models used in the study is available in Appendix 1—table 13.” in Line 475-476 in the Statistical analyses section to indicate this information.

Lines 613-614 - Tweedie is a category of distributions that includes quite a few different options, including Gaussian, Poisson, and Gamma distributions, some of which are normal and some of which are not. So, justifying the use of Tweedie distributions in your model structure doesn't really make sense, and it doesn't really tell me which distribution each model pulled from. Please clarify specifically which distributions you used for each model and why.

We have now added the reason why we used Tweedie distributions by adding the sentence of “There were two models that used the tweedie family (forb and sedge bite rate). We chose to use tweedie because a normal gaussian family model fitted the results poorly” in Line 468-470 in the Statistical analyses section.

We have also included the p-value (power) for forb and sedge bite rate in the Appendix 1—table 13 in the Appendix 1.

Lines 615-618 - Provide citations for R packages described in the text.

We have provided all the related citations for all R packages described in the text, as listed below.

glmmTMB: Brooks, M. E., Kristensen, K., van Benthem, K. J., Magnusson, A., Berg, C. W., Nielsen, A., Skaug, H. J., Maechler, M., & Bolker, B. M. (2017). glmmTMB Balances Speed and Flexibility Among Packages for Zero-inflated Generalized Linear Mixed Modeling. The R Journal, 9(2), 378–400. https://doi.org/10.32614/RJ-2017-066

mgcv: Wood, S.N. (2017). Generalized Additive Models: An Introduction with R (2nd edition). CRC Press. AND Wood, S.N. (2011). Fast stable restricted maximum likelihood and marginal likelihood estimation of semiparametric generalized linear models. Journal of the Royal Statistical Society: Series B (Statistical Methodology), 73(1), 3–36. https://doi.org/10.1111/j.1467-9868.2010.00749.x

DHARMa: Hartig, F. (2022). DHARMa: Residual Diagnostics for Hierarchical (Multi-Level / Mixed) Regression Models. R package version 0.4.6. https://CRAN.R-project.org/package=DHARMa

tidyverse: Wickham, H., Averick, M., Bryan, J., Chang, W., McGowan, L.D., François, R., Grolemund, G., Hayes, A., Henry, L., Hester, J., Kuhn, M., Pedersen, T.L., Miller, E., Bache, S.M., Müller, K., Ooms, J., Robinson, D., Seidel, D.P., Spinu, V., Takahashi, K., Vaughan, D., Wilke, C., Woo, K., & Yutani, H. (2019). Welcome to the tidyverse. Journal of Open Source Software, 4(43), 1686. https://doi.org/10.21105/joss.01686

See these revisions in Line 472-475 in the Statistical Analyses section.

(3) Results

Line 127 - Somewhere in the intro or methods, describe what clipping is and why the pikas do it.

We have provided more details about the clipping behaviors of pikas by adding “Notably, pikas often clip (although do not eat) the wolf poison *S. chamaejasmehas* because its relatively large size hinders predator detection (Fan et al., 1998).” in Line 330-332 in the Methods section.

Line 128 - Change from "preferred" to "consumed greater proportions of"

See the correction in Line 146 in the Results section.

Line 136 - You measured yak weight once a month, so give the result in monthly weight gain rather than daily.

Here we preferred to keep the unit of daily weight gains (converted from monthly ones), as this is the standard presentation for livestock growth performance, also see Fig. 1 in Odadi et al., 2011 Science’s paper.

W. O. Odadi, M. K. Karachi, S. A. Abdulrazak, T. P. Young, Science 333, 1753–1755 (2011).

Lines 138-140 - The linear models, as you described them in the methods (lines 603-620), don't test for hump-shaped relationships. Please update the methods to explain how you tested the density relationship and how you got this interpretation.

The description for Figure 3C here showed estimates from a GAM (family: gaussian), and we did not use a linear model in this figure.

We have now added a new model summary Appendix 1—table 7 for this Figure 3C in the Appendix 1.

Line 145 - I don't think you can claim that the total available forage was more nutritious for yaks, as you picked plants that yaks happened to be chewing along a grazing path. You would need to take samples from a consistent set of plant species at random locations to make this claim.

Sorry for this confusion. We modified “the total available forage” as “the major available forage” here (see Line 172) because we collected the same forage plant species and analyzed their nutrients.

The same as mentioned above, to clarify the sampling methods, we have also revised this point in the Methods section to provide more detail on how forage samples were collected and their quality were analyzed. See these revisions in Line 439-447 in the Methods section.

(4) Discussion

Line 166 - Need to address inconsistency in how you talk about pika density. Here you talk about the impacts of pikas at moderate densities, which I think is a fair claim. Elsewhere, you talk about density-dependence, which I don't think you really measured, given that all your sampling was either in the absence of pikas or within a narrow window of densities that can all be categorized as moderate.

Done! As mentioned above, we have removed the term of “density-dependence” in the whole manuscript, but keep the term of “moderate density” in the Abstract (Line 56-59), Introduction (Line 88-91), Discussion (Line 231-233, the whole paragraph was removed here), and the References section.

Line 176-178 - This is a really cool finding.

Thank you for this positive comment.

Lines 179-181 - You didn't test competition between plant species, and you didn't measure light, soil moisture, or soil nutrients. So you can suggest competition as a potential mechanism, but you can't say definitively that's what is happening.

We now have lowered our tone here as “We speculate that these improvements in food availability and nutrition for yaks may be due to the release of grasses and sedges from competition with the forbs for limiting above- and below-ground resources”.

See the revision in Line 208-211 in the Discussion section.

Lines 184-186 - You did a good job of it here, suggesting a likely potential mechanism at play without claiming it is for sure happening when it hasn't been measured.

Thank you for this positive comment!

Lines 188-199 - Strong paragraph. The impacts of large herbivores on smaller animals are well-studied, but reciprocal impacts are often overlooked.

Thank you for this positive comment!

Lines 201-205 - You didn't test the stress gradient hypothesis because there was no abiotic gradient. You also did not take any measurements during outbreaks, so you cannot claim to have compared low-moderate to outbreak pika densities. I think the paper would be much stronger if you removed the stress gradient hypothesis and instead focused more on the literature around facilitation between mammals of different body sizes, as you do in lines 205-209.

We agreed that our work didn’t specifically design to test the stress gradient hypothesis (SGH) between pikas and yaks, so we have deleted this paragraph here, see Line 231-233 in the Discussion section.

Line 209-215 - Again, you didn't test a competition-facilitation balance because you never tested or demonstrated competition. One of the main strengths of this paper is demonstrating facilitation, so build on that strength rather than referencing things you didn't measure.

The same as mentioned above, we have deleted this paragraph here, see Line 231-233 in the Discussion section.

Lines 218-222 - Not an accurate description of the relationship between herbivore diet and body size. Larger herbivores typically tolerate lower-quality plants in order to consume sufficient calories, but plenty of them do this via mixed feeding. Grazing in large herbivores and livestock is usually due to specifics of the digestive tract (e.g., hindgut fermentation) rather than specifically about body size.

We have deleted this description of the relationship between herbivore diet and body size here. Instead, we have modified this statement as “The coexistence of a diverse of herbivore species with different diet selections and size classes can lead to an “compensatory effect” on grass and forb biomass that helps to maintain a balance and diverse plant community” in the Discussion section.

See these revisions in Line 234-237 in the Discussion section.

Lines 245-251 - Paragraph addresses an important point. Lines 247-249, though, overstate what you measured. There's no measurement of livestock production or biodiversity in the study.

We have replaced the term of “livestock production and biodiversity” with “livestock growth performance” here. See Line 288-298 in the Discussion section.

(5) Figures

Figure 2C-D - This applies to all figures, but you need to plot the best-fit line generated from your model instead of using geom_smooth, which is what these lines look like. You can do this using functions like predict or ggpredict. Using these smoothed lines implied non-linear relationships that you didn't actually test for.

We have redrawn Figures 2C and 2D to use model estimates directly and have included the related model summaries as Appendix 1—table 3 and table 4 in the Appendix 1.

Figure 3B - This figure doesn't show yak weight gain in the presence of pikas. Instead, it shows weight loss when pikas are absent. It's a subtle difference, but very important for interpretation. Yak can maintain weight just fine without pikas as long as Stellera are absent, too. Your results consistently show no Pika x Stellera interactions, but that doesn't match your significance values here. Need to double-check and explain that.

We have revised the descriptions for Figure 3 and 4 in the Results section, by emphasizing that the absence of pikas REDUCED weight gains of yaks, INCREASED toxic plant abundance, and REDUCED the quantity and quality of palatable grasses and sedges. We have revised these sections as below:

Abstract (see Line 51-53)

“Compared to the pika-present treatment, pika removal dramatically increased cover of the poisonous Stellera forbs by two-fold, reducing the abundance and protein content of palatable grasses and sedges, yak foraging efficiency, and yak weight gain by up to 42%.”

Results (see Line 153-167, Line 169-175, Line 184-192)

Also, we did find significant Pika x *Stellera* interactions for yak weight gains, we have provided these details in the Appendix 1—table 5 and table 6 in the Appendix 1.

(6) Recommendations

Lines 245-257 - Would recommend combining the last two paragraphs into one.

We have combined the last two paragraphs, see Line 288-298 in the Discussion section.

Line 530 - Can you replace large with a more precise measure of area?

We are unable to provide a more precise measure of area here, so we have deleted the description of “in a large area”, but we have also added the note of “in the study site” by the end of the sentence to better describe the location of the plots.

See the revision in Line 413 in the Methods section.

Line 541 - Does this mean +/- 7.8 standard deviations? If so, how big is that range in kg?

Here should be “115±7.8 kg”, we have done this correction in Line 392 in the Methods section.

Figure 3 - Would be helpful to use "Stellera/pika present" and "Stellera/pika absent" rather than saying "No Stellera/pika" since you also use the No. abbreviation for numbers a lot in this figure.

We have revised all the related Figures for this issue in Figure 3, 4, and Appendix 1—figure 1,2.

Figure 3F and I - Show letters for significance on these two plots as well, even if it is just a row of a's.

We have redrawn Figure 3F,I to address this issue.

Figure S1 - Applies to all boxplots. Be consistent about showing significance, even if it is a row of a's indicating no difference between treatments.

We have re-drawn Appendix 1—figure 1,2 to address this issue.

Table S1 - Something happened with the line numbers, so they are in the table instead of on the left side.

We have fixed this problem for Appendix 1—table 1 in the Appendix 1.

Table S1 - Applies to all tables. Include the type of model that you ran (including distribution if not Gaussian) in the table legend.

We have provided an summary of all statistical models used in the study in Appendix 1—table 13 in the Appendix 1.

Also, we have added a sentence of “A summary of all statistical models used in the study is available in Appendix 1—table 13.” in Line 475-476 in the Statistical analyses section to indicate this information.

Table S9 - This legend has a good description, including the type of model you used and what you were testing. Apply this more detailed legend to the rest of the tables.

Again! We have provided an summary of all statistical models used in the study in Appendix 1—table 13 in the Appendix 1.

Also, we have added a sentence of “A summary of all statistical models used in the study is available in Appendix 1—table 13.” in Line 475-476 in the Statistical analyses section to indicate this information.
